# Decision analytic cost-effectiveness model to compare prostate cryotherapy to androgen deprivation therapy for treatment of radiation recurrent prostate cancer

**DOI:** 10.1136/bmjopen-2015-007925

**Published:** 2015-10-19

**Authors:** Kathleen A Boyd, Rob J Jones, Jim Paul, Fiona Birrell, Andrew H Briggs, Hing Y Leung

**Affiliations:** 1 Health Economics & Health Technology Assessment, Institute of Health & Wellbeing, University of Glasgow, Glasgow, UK; 2 Cancer Research UK Clinical Trials Unit, Beatson West of Scotland Cancer Centre, Glasgow, UK; 3 Institute of Cancer Sciences, College of Medical, Veterinary and Life Sciences, University of Glasgow, Bearsden, Glasgow, UK; 4 Department of Urology, NHS Greater Glasgow and Clyde, Glasgow, UK; 5 Beatson Institute for Cancer Research, Bearsden, Glasgow, UK

**Keywords:** radiation recurrent prostate cancer, cost-effectiveness analysis, androgen deprivation therapy, Prostate disease < UROLOGY

## Abstract

**Objective:**

To determine the cost-effectiveness of salvage cryotherapy (SC) in men with radiation recurrent prostate cancer (RRPC).

**Design:**

Cost-utility analysis using decision analytic modelling by a Markov model.

**Setting and methods:**

Compared SC and androgen deprivation therapy (ADT) in a cohort of patients with RRPC (biopsy proven local recurrence, no evidence of metastatic disease). A literature review captured published data to inform the decision model, and resource use data were from the Scottish Prostate Cryotherapy Service. The model was run in monthly cycles for RRPC men, mean age of 70 years. The model was run over the patient lifetime, to assess changes in patient health states and the associated quality of life, survival and cost impacts. Results are reported in terms of the discounted incremental costs and discounted incremental quality-adjusted life years (QALYs) gained between the 2 alternative interventions. Probabilistic sensitivity analysis used a 10 000 iteration Monte Carlo simulation.

**Results:**

SC has a high upfront treatment cost, but delays the ongoing monthly cost of ADT. SC is the dominant strategy over the patient lifetime; it is more effective with an incremental 0.56 QALY gain (95% CI 0.28 to 0.87), and less costly with a reduced lifetime cost of £29 719 (€37 619) (95% CI −51 985 to −9243). For a ceiling ratio of £30 000, SC has a 100% probability to be cost-effective. The cost neutral point was at 3.5 years, when the upfront cost of SC (plus any subsequent cumulative cost of side effects and ADT) equates the cumulative cost in the ADT arm. Limitations of our model may arise from its insensitivity to parameter or structural uncertainty.

**Conclusions:**

The platform for SC versus ADT cost-effective analysis can be employed to evaluate other treatment modalities or strategies in RRPC. SC is the dominant strategy, costing less over a patient's lifetime with improvements in QALYs.

**Trial registration number:**

This economic analysis was undertaken as part of the CROP RCT study ISRCTN:72677390; it was a pre-trial economic model developed and analysed during the pre-results stage of the RCT.

**Strengths and limitations of this study**

- Androgen deprivation therapy (ADT) is often overutilised in patients who could be potential candidates for salvage local therapy. We developed a decision model informed by the best available evidence on the benefits, quality of life and costs of treatment with salvage cryotherapy (SC) and ADT to inform a cost-effectiveness analysis. We adhered to good practice guidelines and the National Institute for Health and Care Excellence (NICE) reference case, reporting outcomes as discounted incremental cost per quality-adjusted life year gained.
- To our knowledge, this is the first economic analysis comparing the use of SC against ADT. Several evaluations have explored the cost-effectiveness of ADT in advanced/metastatic prostate cancer, but none in the radiation recurrent prostate cancer (RRPC) population, and none on SC in RRPC.
- To date, there have been no direct head-to-head comparisons of cryotherapy and ADT in a randomised controlled trial; however, the outcomes from published reports from multiple centres in the USA and Europe justify the significance and importance of cryotherapy as an alternative to ADT in the RRPC population.
- This study found a lack of direct head-to-head comparison data for use in the model, and therefore the main assumptions employed in the model were conservative and used to work against cryotherapy. The analysis was undertaken using probabilistic sensitivity analysis to account for uncertainty in the parameter inputs.
- Cryotherapy is an important local treatment option for RRPC sufferers. The UK decision body NICE drew attention to a lack of evidence on cryotherapy as a treatment for recurrent prostate disease in 2008, and recommended it only be used in clinical trials until further economic and clinical evidence was established. Hence, the methodology and data presented in this report are of direct relevance to decision-making bodies.

## Introduction

Radiation recurrent prostate cancer (RRPC) is a well-established global issue, yet receives little attention from the research community and is supported by inadequate data to inform evidence-based decisions.^1^ When considering a conservative estimate of 25% disease relapse following primary radiation therapy, over 13 600 new cases of RRPC are expected each year in the USA,^2^ with approximately 81 000 across Europe.^3^ This translates into a substantial financial burden on healthcare services, with the long-term cost of prostate cancer care (diagnosis, treatment and follow-up) for 5 years in European countries ranging from €198 million (Spain) to €618 million (France).^4^ Given the scale of financial burden incurred by prostate cancer, a clear and robust platform to evaluate the cost-effectiveness of current and emerging treatment options is necessary.

Androgen deprivation therapy (ADT) is a palliative treatment with well-documented side effects and health risks.^5^ Deferred or immediate ADT remains the commonest strategy used for RRPC sufferers^6–8^ and is often overutilised in patients who could be potential candidates for salvage local therapy. This reflects the lack of high-quality, mature supportive evidence from prospective multicentre studies on alternative treatments, including salvage surgery (prostatectomy, cystoprostatectomy)^9^ ^10^ and local ablative approaches (cryotherapy, focal brachytherapy, high-intensity focused ultrasound). Salvage prostate cryotherapy is a viable option with an acceptable efficacy and toxicity profile.^11–15^ While the debate continues among proponents of different RRPC therapies, cost-effectiveness analyses are integral and essential to policy-making considerations. We set out to develop a platform for decision analytic cost-effectiveness evaluation by comparing ADT and salvage cryotherapy (SC) for patients with RRPC.

## Materials and methods

Decision analytic modelling was used to evaluate the potential cost-effectiveness of SC in comparison to ADT in the RRPC population (biopsy-proven local recurrence with no evidence of metastatic disease).^16^ We adhered to good practice guidelines and the National Institute for Health and Care Excellence (NICE) reference case,^17^ reporting outcomes as discounted incremental cost per quality-adjusted life year (QALY) gained (cost year 2014) from the perspective of the UK National Health Service (NHS). The UK decision threshold of £20 000–£30 000/QALY will be used to determine cost-effectiveness.^17^ The economic model was designed, developed and populated based on published literature and in accordance with clinical practice. A literature review was undertaken (MEDLINE, EMBASE, Cochrane Library—NHSEED, HTA, DARE—and the CEA Registry from 1990 to August 2014) to identify data on patient survival and disease progression following ADT and SC in the RRPC population. Economic evaluations of ADT or SC in this patient group were also incorporated. Literature was required to inform specific model parameters, such as utility estimates for patient quality of life. Further details of the literature search are available in the online supplementary material. Estimates of resource use were obtained from the Scottish Prostate Cryotherapy Service^18^ and guided by clinical experts in the field. Unit costs were obtained from reference sources such as the British National Formulary,^19^ Information Services Division^20^ and the Department of Health^21^ (see online supplementary information).

### Model structure and parameters

**Figure 1:**
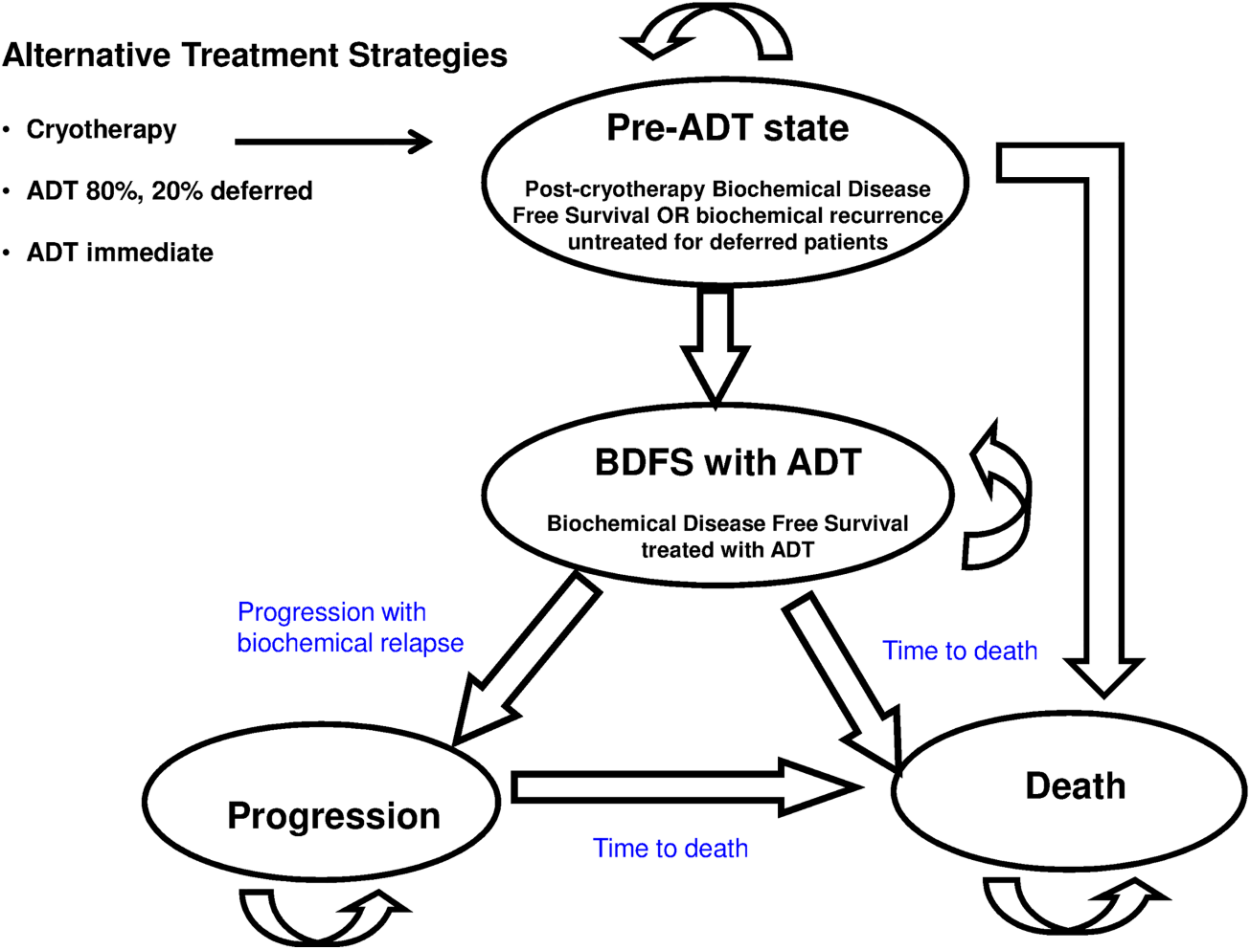
Alternative treatment strategies and Markov model (ADT, androgen deprivation therapy; BDFS, biochemical disease-free survival).

Figure 1 details the alternative treatment strategies and Markov model, with transition between states limited to the direction illustrated by arrows. A patient entering the model will have previously received primary radiotherapy (either brachytherapy or external beam) for his prostate cancer and subsequently developed biochemical relapse with histologically confirmed local recurrence without detectable metastases.^16^ Importantly, he will be deemed eligible for a randomised controlled trials (RCTs) as described in ref. 16, thus providing a valid basis for comparison between different treatment options. Such a patient can be treated with SC, ADT or a third strategy where 80% of patients receive ADT immediately and 20% have deferred ADT.^6–8^ It is unlikely that in practice, all patients would immediately receive ADT, and therefore this third strategy (ADT with 20% deferred) was included to reflect current practice.^6–8^ The Markov model comprises four states: ‘pre-ADT’ which for post-SC patients is biochemical disease-free survival (BDFS), ‘BDFS with ongoing ADT’, ‘Progression’ and ‘Death’. Cryotherapy-associated adverse events (urinary incontinence, obstructive urinary symptoms/retention, lower urinary tract symptoms, perineal pain, haematuria, urethral stricture and fistula) will typically manifest within 3 months post-treatment,^22^ and are assigned an additional cost and quality of life decrement for this time period. Patients with troublesome ADT-induced hot flushes may require additional antiandrogen treatment. Patients who received SC will enter the Markov model with the nadir prostate-specific antigen (nPSA) level achieved, with nPSA <1 ng/mL being a good prognostic marker.^13^ ^22^ At the end of each monthly cycle, SC patients can experience death, remain in the pre-ADT state or develop biochemical recurrence^13^ triggering first-line ADT (with patients entering the lower BDFS state). In the ADT arm, all patients enter the model in the BDFS with ADT state, receiving monthly treatment with goserelin as clinically recommended.^8^ ^23^ The third strategy accounts for a minority (20%) of patients who are assumed to have deferred ADT;^8^ these patients enter the model in the ‘pre-ADT’ state with a ‘do nothing’ approach to their biochemical recurrence. The remaining 80% begin the Markov model in the BDFS with ADT state. Here patients can experience death, remain stable or experience disease progression with biochemical relapse +/− metastases. In the Progression state, 40% of patients will have metastatic disease at any one time.^24^ Those patients in the Progression state will receive second-line ADT as clinically indicated (monthly treatment with abiraterone^i^ for metastatic progression, or with leuprorelin acetate for non-metastatic progression).

The base-case model begins with a cohort of 1000 patients with RRPC (biopsy-proven local recurrence with no evidence of metastatic disease)^16^ who have a mean age of 70,^13^ ^14^ and runs in monthly cycles for a time horizon of 36 years (432 cycles), until everyone in the cohort has died. This model is run for each of the treatment groups using the intervention-specific costs, disease-free survival (DFS) estimates, age-specific population mortality rates and utility estimates, so as to calculate total costs, life years gained (LYG) and QALY outcomes for each treatment group. Costs and QALYs were discounted at 3.5% as recommended for the NICE reference case.^17^

Table 1 details the main parameters used in the model, their SEs and the distribution used in the probabilistic analysis. The resource use parameters, their SEs and unit costs are detailed in table 2. The online supplementary information document provides further details of the Markov model, and online supplementary table S2 details a summary of key model assumptions.

**Table 1:** Base-case model parameters

Parameter	Point estimate	SE	Probabilistic distribution	Data source
Mean age (years)	70	NA	NA	13 and 14
Model lifetime (years)	36	NA	NA	13 and 14
Discount rate (costs and outcomes)	0.035	NA	NA	17
Proportion-confirmed metastases in progression	0.4	0.08	β	AA^24^
Transition probabilities (monthly)				
Cryotherapy to ADT	Spline regression fitted to Kaplan Meier DFS curve			13
Spline model hazard intercept	−4.58	0.408	Normal	Spline PH regression^13^
Spline model hazard s0	1.01	0.096	Normal	Spline PH regression^13^
Spline model hazard s1	0.303	0.068	Normal	Spline PH regression^13^
Deferred ADT to ADT	0.014	0.005	β	36
ADT to progression	0.016	0.004	β	36
Relative risk ADT to progression post-SC	1.5	1.38	Log normal	AA
UK mortality rates: males, age specific	2010 UK interim life tables			40
Prostate cancer mortality: age specific	2010 UK prostate cancer mortality rates			3
Adverse event probabilities				
Fistula	0.05	0.010	β	22 33 and 35
Incontinence	0.12	0.024	β	22 33 and 35
Retention	0.07	0.014	β	22 33 and 35
Lower urinary tract symptoms	0.14	0.028	β	22 33 and 35
Perineal pain	0.04	0.008	β	22 33 and 35
Haematuria	0.05	0.01	β	22 33 and 35
Urethral stricture	0.10	0.02	β	22 33 and 35
Hot flushes	0.10	0.02	β	Clinical expertise^32^
Cost				
Cryotherapy*	£8509	NA	NA	18 and 20
First-line ADT per cycle*	£83	NA	NA	19
Second-line ADT per cycle*: confirmed metastases†	£2930	NA	NA	19
Second-line ADT per cycle*: non-metastatic†	£95	NA	NA	19
Fistula	£4039	£808	γ	21
Incontinence	£2170	£434	γ	21
Retention	£3963	£793	γ	21
Lower urinary tract symptoms	£10.47	NA	NA	19
Perineal pain	£5.16	NA	NA	19
Haematuria	2445	£489	γ	21
Urethral stricture	£3836	£767	γ	21
Hot flushes (per cycle)	£5.54	NA	NA	19
Utility				
Disease-free survival	0.774	0.02	β	41
Progression: metastases	0.42	0.06	β	36 and 42
Progression: non-metastatic	0.68	0.022	β	41
Disutility for fistula	0.15	0.03	β	AA/clinical experts

*Not probabilistic, but cost varies depending on probabilistic resource use parameters for delivery; see table 2.

†Depends on the proportion of patient in Progression state who have confirmed metastases.

ADT, androgen deprivation therapy; AA, author assumption made in conjunction with clinical experts; ICER, Incremental Cost-effectiveness Ratio; PH, proportional hazards; SC, salvage cryotherapy.

**Table 2:** Costs: unit costs, resource use and total costs

Cost item	Unit cost	Resource use	SE	Total cost	Source
Cryotherapy surgery					
Ultrasound	£47.84	1	NA	£47.84	18 and 20
Surgeon time (consultant)	£142.00	2.5 h	0.25*	£355.00	20
Theatre (including staff, appliances, drugs, etc)	£1125.00	2 h	0.2*	£2250.00	20
Additional theatre cost(Argon, Helium, balloon and guidewire)	£260.61	1	NA	£260.61	18
Freezing needles (per kit including 2 freezing and 8 temp needles)	£5052.17	1 kit	NA	£5052.17	18
Overnight stay in hospital	£237.34	2 nights	0.2*	£474.68	21
Catheter fitting and removal (40 min in total)	£53.00	0.66 h	0.066*	£35.33	20
				**£8475.64**	
Cryotherapy medication					
Antibiotics: ciprofloxacin 10 tablets 250 mg twice daily×3 days	£0.93	1	NA	£0.93	18 and 19
Painkillers: acemetacin (Emflex) 60 mg, 90 capsule pack	£28.20	1	NA	£28.20	18 and 19
α-blockers: tamsulosin, 400 mg daily 30 tablet pack	£4.42	1	NA	£4.42	18 and 19
				**£33.75**	
Total cost per cryotherapy patient				**£8509.39**	
Cryotherapy acute adverse events					
Incontinence: urinary incontinence with intermediate complications	£2170	1	£434	£2170	20
Lower urinary tract symptoms: tamsulosin 30 tablets, 400 µg	£10.47	1	NA	£10.47	18 and 19
Perineal pain: ibuprofen 400 mg 30 tabs, 4× daily	£3.61	1.42	NA	£5.13	18 and 19
Haematuria: elective inpatient catheter	£2445	1	£489	£2445	20
Urethral stricture: major open urethra procedure	£3836	1	£767	£3836	20
Retention†			£793	£3963	20
Fistula‡			£808	£4039	20
First-line ADT regime					
Bicalutamide (10 days prior to LHRH—first month only)	£5.54	1	NA	£5.54	18 and 19
Goserelin: LHRH	£65.00	1	NA	£65.00	18 and 19
Bicalutamide (treatment for hot flush side effect)	£5.54	0.1	NA	£0.55	18
Delivery of goserelin (practice nurse)	£53.00	0.33 h	0.066*	£17.67	18 and 19
Total cost for first-line ADT first cycle				**£88.76**	
Total cost per first-line ADT cycle (every 28 days)				**£83.22**	
Second-line ADT (confirmed metastases)					
Abiraterone tablets (monthly cost)	£2930.00	1	NA	**£2930.00**	19
Second-line ADT (rising PSA non-metastatic progression)					
LHRH agonist: leuprorelin acetate	£75.24	1	NA	£75.24	18 and 19
Flutamide for proportion who have hot flush side effect	£25.37	0.1	NA	£2.54	18 and 19
Delivery (practice nurse)	£53.00	0.33 h	0.066*	£17.67	18 and 19
Total cost second-line ADT non-metastatic disease per cycle				**£95.44**	
Mean cost of second-line ADT in progression state per cycle§				**£1229.27**	

Bold typeface indicates total costs for the main cost items.

*γ Distributions used for resource use parameters in the probabilistic analysis.21a

†Depends of proportion-confirmed metastases and non-confirmed. Base-case model 40% metastases (SE 0.08); see table 1.

‡Retention: elective inpatient catheter plus cost of transurethral resection of prostate for 50% of retention patients.

§Fistula: 4 hospital consultations, MRI plus 50% need colostomy or repair surgery.

ADT, androgen deprivation therapy; LHRH, luteinising hormone releasing hormone; PSA, prostate-specific antigen.

### Uncertainty and sensitivity analyses

Probabilistic sensitivity analysis was undertaken using Monte Carlo simulation (10 000 iterations) to reflect uncertainty in the input parameter estimates.^25^ The 10 000 incremental cost and QALY outcomes are plotted on a cost-effectiveness plane to illustrate uncertainty in the model results. The cost-effectiveness acceptability curve (CEAC) is presented to illustrate the probability of each strategy being cost-effective at different willingness to pay thresholds.

Five scenario analyses were undertaken to explore the impact of varying some of the base-case assumptions.^26^ Scenario 1 explores the impact of abiraterone by assuming this expensive treatment is not available for metastatic progression, instead all patients receive the much cheaper leuprorelin acetate treatment (£97 per month). Scenario 2 applies a greater relative risk of Progression from BDFS with ADT for patients previously treated by cryotherapy. This is a key uncertainty in the model as there have been no RRPC trials directly comparing SC with ADT, and therefore no evidence to suggest whether the risk of progression with ADT for postcryotherapy-relapsed disease would be the same or greater when compared with patients with RRPC receiving ADT without SC. Given this uncertainty, the base-case analysis took a pessimistic outlook for SC and applied a relative risk of 1.5; scenario 2 applies a greater relative risk of 2, increasing the hazard of progression in the SC arm and ensuring that the pre-ADT state in the model does not implicitly bias towards the SC arm in terms of longevity. A third scenario used alternative data^15^ for risk of recurrence following SC. In the final two scenarios, patient heterogeneity in the model was explored by re-running the model for a mean patient age of 60 and 80 years, respectively.

As detailed in the online supplementary information, sensitivity analyses were also undertaken to ensure the base-case spline distribution used for risk of recurrence with SC was robust^27^ and the best fit to the Kaplan-Meier data.^13^

## Results

SC was on average £38 763 (€49 067) cheaper (95% CI −63 535 to −16 175) than ADT and £29 719 (€37 619) cheaper than the ADT 20% deferred strategy over a patient's lifetime, and was more effective with a mean QALY gain of 0.68 (95% CI 0.4 to 1.04) and 0.56 (95% CI 0.28 to 0.87), respectively (table 3A). There was little difference in terms of life year gains between the strategies, but when adjusted for quality of life, SC is clearly the dominant strategy. The ADT immediate strategy is dominated by both the SC and ADT 20% deferred strategies, and as it is unlikely in practice that no patients would have deferred treatment,^8^ the results and scenario analysis will from now onwards compare SC to the ADT 20% deferred strategy.

**Table 3:** Results

		Mean			Inc cost	Inc QALYs		Probabilistic CE	Cost neutral
		Cost	LYs	QALYs	(95% CI)	(95% CI)	ICER	£30 000	Time point
(A) Base-case Procedure									
Cryotherapy (SC)		£62 150	10.62	7.59	NA	NA	NA	1.0	
ADT 20% deferred		£91 869	10.58	7.03	£29 719	−0.56	SC dominates	0.0	3.5 years
					(£9243 to £51 985)	(−0.87 to −0.28)			
ADT immediate		£100 914	10.57	6.91	£38 763	−0.68	SC dominates	0.0	3 years
					(£16 175 to £63 533)	(−1.04 to −0.4)			
(B) Scenario outcomes									
Scenario									
(1) No abiraterone	ADT 20%	£10 529	10.58	7.04	NA	NA		0.0	Never
Low-cost second ADT	SC	£14 820	10.62	7.59	£4291	0.55	£7801	1.0	(>36 years)
					(£1577 to £6253)	(0.29 to 0.86)			
(2) Relative risk=2	ADT 20%	£91 894	10.58	7.02	NA	NA		0.001	4 years
Progression post-SC	SC	£62 149	10.62	7.54	−£29 745	0.51	SC dominates	0.997	
					(−£53 847 to −£9320)	(0.22 to 0.86)			
(3) DFS data post-SC	ADT 20%	£91 959	10.58	7.03	NA	NA		0.029	3.5 years
Wenske *et al*^15^	SC	£62 156	10.63	7.66	−£29 803	0.63	SC dominates	0.971	
					(−£80 269 to £2597)	(0.05 to 1.19)			
(4) Mean age 60 years	ADT 20%	£142 629	14.49	9.40	NA	NA		0.0	3.5 years
	SC	£93 410	14.53	10.18	−£49 219	0.77	SC dominates	1.0	
					(−£80 549 to −£22 615)	(0.4 to 1.18)			
(5) Mean age 80 years	ADT 20%	£46 997	6.67	4.58	NA	NA		0.0	4 years
	SC	£32 362	6.70	4.90	−£14 635	0.33	SC dominates	1.0	
					(−£27 504 to −£7382)	(0.17 to 0.51)			

ADT, androgen deprivation therapy; CE, cost-effective; DFS, disease-free survival; Inc, incremental; LYs, life years; QALYs, quality-adjusted life years; SC, salvage cryotherapy.

**Figure 2:**
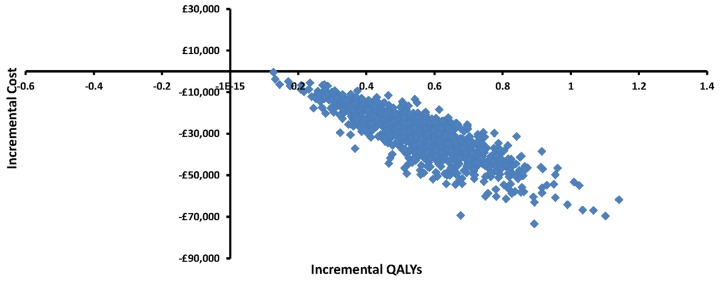
Cost-effectiveness plane for SC compared with ADT 20% deferred (ADT, androgen deprivation therapy; QALYs, quality-adjusted life years; SC, salvage cryotherapy).

**Figure 3:**
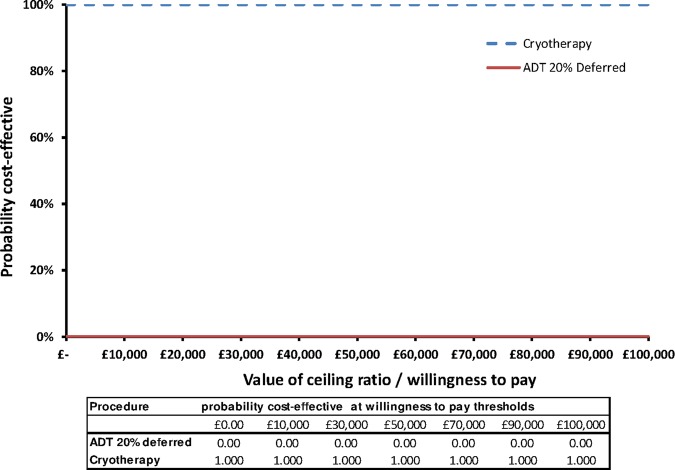
Cost-effectiveness acceptability curves for salvage cryotherapy and androgen deprivation therapy (ADT) 20% deferred.

Figure 2 plots the 10 000 incremental cost and QALY outcomes from the probabilistic analysis on the cost-effectiveness plane. All of the points fall in the south-eastern quadrant, reinforcing SC as a dominant treatment strategy for RRPC with cost-savings and improved QALYs. There is little uncertainty in the cost-effectiveness decision over a wider range of willingness to pay thresholds, as demonstrated in the CEAC (figure 3). Considering the NICE threshold of £30 000/QALY,^17^ SC has a 100% probability to be cost-effective (figure 3).

**Figure 4:**
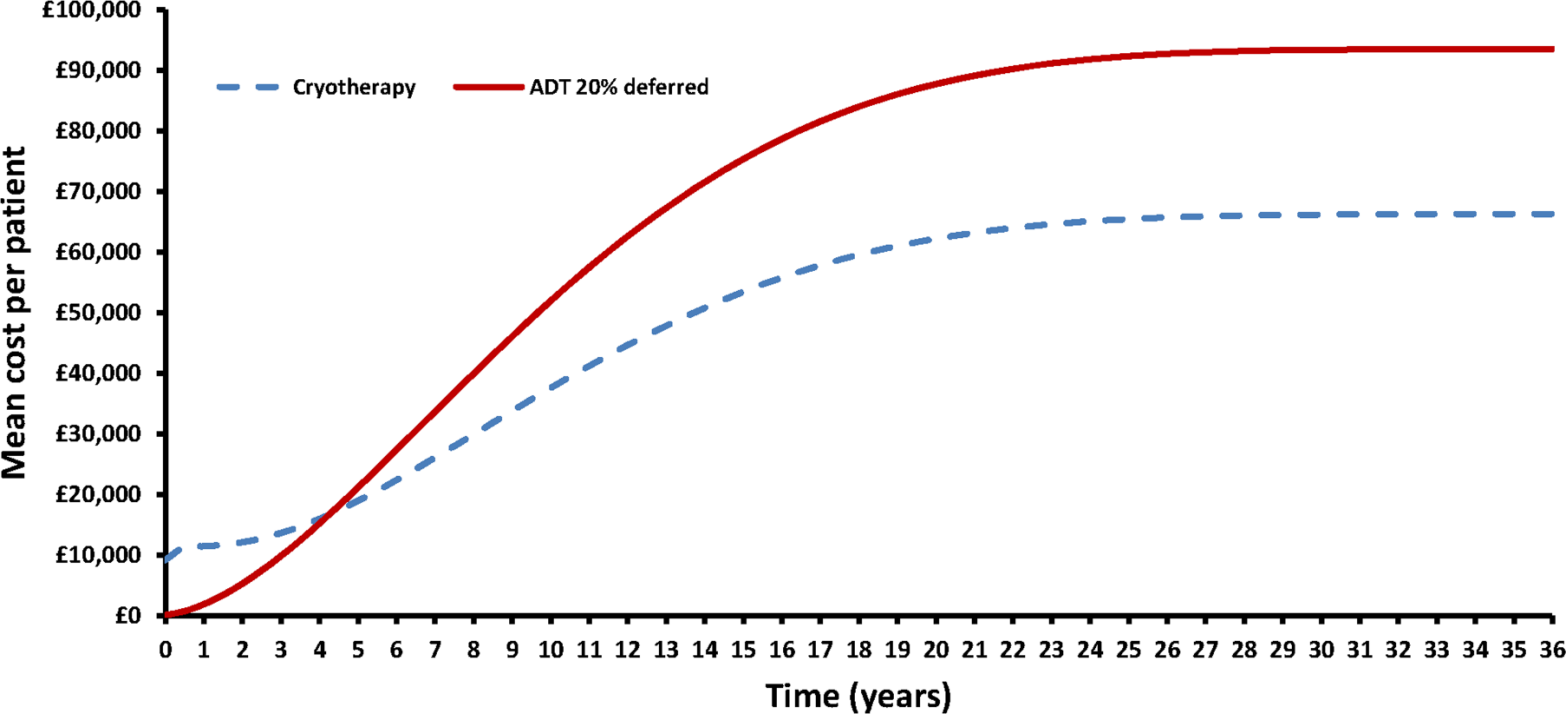
Mean cost per patient over model lifetime (ADT, androgen deprivation therapy).

Although SC has an expensive upfront treatment cost of £8509 (€10 771), by resetting the ‘PSA clock’, it delays and may avoid the ongoing monthly treatment with ADT. Figure 4 illustrates how the mean cost per patient varies in each arm over time. The two curves intersect at 3.5 years, which signifies the cost neutral point, where the upfront cost of SC (plus any subsequent cumulative cost of ADT) per person equates the cumulative cost per person in the ADT arm.

Sensitivity analyses (table 3B) illustrate that the outcomes remain mostly unchanged to modifications in key model assumptions; and where outcomes do change, the cost-effectiveness decision remains in favour of cryotherapy. SC remains the dominant strategy under all but scenario 1, with a 100% probability of being the cost-effective strategy at a threshold of £30 000/QALY. Scenario 1, which assumed a lower monthly cost for second-line ADT (£95/month, no abiraterone for metastatic treatment) resulted in the SC arm costing an additional £4291 (€5432) over the patient lifetime, with a QALY gain of 0.55. In this scenario, the model does not reach a cost neutral point; however, the ICER of £7801/QALY (€9875/QALY) is well below the UK decision threshold of £20 000–£30 000/QALY.

## Discussion

We developed a decision model informed by the best available evidence on the benefits, quality of life and costs of treatment with SC and ADT for patients with RRPC. The model predicted that cryotherapy would be cost-saving compared with ADT, and would offer improvements in QALYs gained. Upfront treatment with cryotherapy delayed, and in some cases avoided, subsequent treatment with ADT, giving a cost-neutral time of 3.5 years (the time it takes for a patient in the ADT arm to equate the cost of a patient treated by SC). Cryotherapy was found to be the dominant strategy over a wide range of cost-effectiveness thresholds and under a variety of scenario analyses.

The QALY decision threshold for cost-effectiveness is subject to debate internationally and varies from country to country;^28–30^ however, as shown in the CEAC in figure 3, setting a lower, or higher threshold equivalent to US$50 000^29^ or the €80 000 as suggested in the Netherlands,^30^ would not alter the conclusions of our analysis.

Several economic evaluations have been undertaken to explore the cost-effectiveness of ADT in advanced/metastatic prostate cancer, but none in the RRPC population, and none on SC in RRPC (biopsy-proven local recurrence with no evidence of metastatic disease); so to our knowledge, this is the first cost-utility analysis modelling and comparing the use of SC against ADT.

The literature search identified only nine papers^11–14^ ^31–35^ detailing DFS evidence for SC in a RRPC population. All were either retrospective analyses at clinical urology units, online data registries or prospective case studies, without a direct comparator or control. Given this lack of evidence, we used DFS data from Williams *et al*,^13^ which remains a landmark report for patient outcome following SC with the longest follow-up captured in Kaplan-Meier curves for DFS. Previous cost-effectiveness analyses in advance prostate cancer have simply modelled progression as a constant hazard using point estimates from published data to determine transition probabilities.^36^ Our analysis used Kaplan-Meier data, and explored a variety of parametric distributions to determine progression hazard for cryotherapy. It may be argued that existing SC studies have been conducted in highly select patient groups giving overoptimistic results; therefore, our base-case model incorporated an increased relative risk of progression from ADT for postcryotherapy patients, to counteract any ‘overoptimism’ for cryotherapy. Scenario 2 exaggerated this risk further, but even so, SC remained cost-effective. Additionally, scenario 3 used alternative data^15^ for the hazard of biochemical relapse postcryotherapy. Even though there has been no direct head-to-head comparisons of cryotherapy and ADT in the context of a RCT,^16^ the outcome from published reports based on work in multiple centres in the USA and in Europe^11–14^ ^31–35^ justify the significance and importance of cryotherapy as an alternative to ADT in the RRPC population. ADT is often overutilised in patients who could be potential candidates for salvage local therapy or other (whole gland or focal) ablative therapy, and therefore this paper synthesises the existing evidence to provide necessary information to inform a cost-effectiveness analysis. The majority of assumptions employed in the model were conservative and used to work against cryotherapy. All assumptions have been fully described in the online supplementary table S2, and have been applied based on the best available evidence and in consultation with clinical experts on the CROP trial team.^16^ Regardless of the alternative assumptions or strategy employed, cryotherapy remained the cost-effective option.

The decision model in this paper has shown that although cryotherapy has an expensive upfront treatment cost, within 3.5 years the mean cost per patient is equivalent to the mean cost of patients treated with ADT only.

ADT has a much lower, yet ongoing, cost of approximately £83 (€105) per month (goserelin every 28 days) which continues until a patient dies, or progresses and then incurs the expensive second-line ADT regimen (abiraterone for confirmed metastases, leuprorelin for non-confirmed metastatic progression). Over the remaining patient lifetime, the cost of ADT accumulates. As patients in the SC arm only receive first-line ADT on biochemical recurrence following cryotherapy, this further delays the need for second-line ADT when compared with patients receiving ADT. It is this avoidance and delay in both first-line and second-line ADT that leads to substantial cost-savings of approximately £29 719 (€37 619) per patient in the SC arm. Second-line ADT treatment is significantly more expensive than first-line ADT costing approximately £1229 (€1555) per patient month (assuming 40% of patients are treated for confirmed metastatic progression^24^ with abiraterone at £2930/month, and the remaining 60% for non-metastatic progression at £95/month).

Scenario 1 showed that it is the high cost of abiraterone which leads to the cost-saving advantages of SC in the base-case model. When costly abiraterone treatment is removed (scenario 1), SC is found to be more expensive than the ADT 20% deferred strategy, but would still be cost-effective with an ICER of £7801 (€9874).

The current clinical practice for patients with relapsed disease following salvage prostate cryotherapy (and indeed for any other local ablative therapy) is androgen ablation therapy. For these patients, the treatment pathway will mirror that of patients who receive ADT as second-line treatment, without cryotherapy. Therefore, treatment with ADT is the expected clinical patient journey for patients failing cryotherapy. As the model outcomes show, patients who receive cryotherapy will essentially have their ‘PSA clock’ reset. A key issue from this analysis is that the model identifies the magnitude of this ‘PSA clock’ reset required to adequately impact on the cost-effectiveness of cryotherapy when compared with the most commonly used treatment, that is, ADT. It is also important to note that in terms of LYG, there is little difference between SC, ADT and ADT 20% deferred strategies. The advantage of SC appears to be in delaying, and in some cases avoiding, progression of disease, through resetting the ‘PSA clock’, which is particularly important in terms of metastatic progression where patients are more likely to incur much higher treatment costs and lower quality of life.

With regard to adverse events, previous studies of cryotherapy and ADT have found that short-term adverse events have very little effect on overall/long-term quality of life;^37^ however, they may incur considerable additional costs in patient treatment, so it is important to capture any cost or quality of life impacts of any acute adverse events and how these may impact on model outcomes. Therefore, the model allowed for patients in the SC cohort to experience the seven most common SC-related adverse events (incontinence, retention, lower urinary tract symptoms, perineal pain, haematuria, urethra stricture and fistula), utilising the worst case estimates reported in recent literature^22^ ^33^ ^35^ to ensure the base-case analysis was pessimistic towards cryotherapy. Persistent erectile dysfunction would be experienced by the majority of patients who have RRPC before undertaking cryotherapy or beginning ADT, as a result of prior treatment,^6^ which is accounted for in the baseline utility level for all cohorts (as detailed in online supplementary material). While a small proportion of patients may still be potent when entering the model, and therefore have a slightly higher quality of life than those who are impotent at baseline,^38^ the proportion of patients with erectile dysfunction would be the same in each arm of the cohort model, and therefore stratifying the baseline population by impotence would not make any difference to incremental gains and losses in QALY outcomes. The age-adjusted utility value used for the cohort population in each arm reflects a mid-way quality of life point between potent patients and those suffering from impotence. Tables 1 and 2 detail the probability estimates and additional costs for treating SC-related events (incontinence, retention, lower urinary tract symptoms, perineal pain, haematuria, urethra stricture and fistula). Our model results are consistent with previous studies^36^ ^37^ in that these acute SC-related adverse events which are only experienced for the first few months have little impact on long-term quality of life and QALY outcomes.^36^ ^37^ The high upfront cost of treating these adverse events (see table 2) also has no significant impact in the longer term model outcomes for SC. This is most likely due to (1) the relatively low incidence of second surgical procedures in patients receiving SC (see table 1) and (2) also because the cost of SC-related adverse events are overshadowed by the lifetime cost-savings and quality of life benefits of SC through delaying, and in some cases avoiding, progression of disease, through resetting the ‘PSA clock’. As with all model-based economic evaluations, a limitation of this analysis is that the model and assumptions are based on retrospective data, and therefore subject to the quality of this data.^39^ In response to this limitation, the majority of assumptions (see online supplementary table S2) employed in the model were conservative and used to work against cryotherapy. Cryotherapy is an important local treatment option for RRPC sufferers. The UK decision body NICE drew attention to a lack of evidence on cryotherapy as a treatment for recurrent prostate disease in 2008, and recommended it only be used in clinical trials until further economic and clinical evidence was established.^8^ Hence, methodology and data presented in this report are of direct relevance to decision-making bodies. This is particularly relevant as the CROP RCT study (ISRCTN: 72677390, http://www.controlled-trials.com/ISRCTN72677390)^16^ comparing SC and androgen ablation has recently been stopped due to patients declining randomisation opting for SC as a preferred treatment.

The intention of this model was to perform the cost-effectiveness analysis on the most common treatment options in RRPC. The platform developed in this paper for SC versus ADT cost-effective analysis can be employed to evaluate other treatment modalities or strategies in RRPC. This paper will be critical to add to the debate regarding overutilisation of ADT and underutilisation of salvage local therapy in the RRPC population, and it is intended that this model can contribute to future service planning for patients with RRPC.

Our report has synthesised the existing evidence in a probabilistic analysis and shown that, despite a high upfront treatment cost, SC substantially reduces the cost of treating patients with RRPC over their lifetime by delaying, and in some cases avoiding, ADT. We showed that cryotherapy is a dominant treatment strategy when compared with upfront ADT and should therefore be reconsidered by decision-making bodies as a viable treatment option for patients with RRPC.

## Conclusion

SC is a dominant strategy in comparison to ADT, costing less over a patient's lifetime while offering improvements in QALYs. Probabilistic analysis (to account for uncertainty in the model parameters) showed SC to be the dominant strategy over a wide range of thresholds. Under alternative model assumptions, the cost-effectiveness decision remains in favour of cryotherapy.

## Footnotes

i Enzalutamide is an alternative to abiraterone, but the price and duration difference between them is negligible, so substituting abiraterone for enzalutamide in the model would not impact on the costs or the outcomes.
