# Correction: *Decision analytic cost-effectiveness model to compare prostate cryotherapy to androgen deprivation therapy for treatment of radiation recurrent prostate cancer*

**DOI:** 10.1136/bmjopen-2015-007925corr1

**Published:** 2016-08-03

Boyd KA, Jones RJ, Paul J, *et al.* Decision analytic cost-effectiveness model to compare prostate cryotherapy to androgen deprivation therapy for treatment of radiation recurrent prostate cancer. *BMJ Open* 2015;5:e007925. This paper has been resupplied with the correct license statement.

